# Frequency of hearing loss among medical students using electroacoustic device

**DOI:** 10.12669/pjms.38.3.4927

**Published:** 2022

**Authors:** Saba Asghar, Hurtamina Khan, Saima Parveen, SM Tariq Rafi

**Affiliations:** 1 Saba Asghar, Final Year Medical Student Jinnah Sindh Medical University, Karachi, Pakistan; 2 Hurtamina Khan, FCPS. Assistant Professor, ENT and Head & Neck Surgery Jinnah Postgraduate Medical Centre, Jinnah Sindh Medical University, Karachi, Pakistan; 3 Saima Parveen, Final Year Medical Student, Jinnah Sindh Medical University, Karachi, Pakistan; 4 S. M. Tariq Rafi, FCPS, FRCS. Professor Emeritus of ENT, Vice Chancellor, Jinnah Sindh Medical University, Karachi, Pakistan

**Keywords:** Personal listening devices, Pure Tone Audiometry, medical students, sensorineural hearing loss, noise induced hearing loss, recreational noise

## Abstract

**Objective:**

To determine frequency of hearing loss among medical students using electroacoustic devices like hands free, headphone etc. through Pure Tone Audiometry.

**Methods:**

This cross-sectional study was conducted among students at JSMU from December 2019 till February 2020. Ethical approval was obtained against Ref: JSMU/IRB/2019/-215. Calculated sample size was 194. Non-probability convenience sampling technique was employed. Students were invited to ENT OPD JPMC, Karachi. After informed consent, sociodemographic and electroacoustic device usage history was recorded. PTA was performed at octave frequencies for air (0.25-8kHz) and bone conduction (0.5kHz-4kHz). WHO grading of hearing impairment was used. Statistical analyses carried through IBM SPSS. Chi square test, Fischer exact test and independent *t* test were applied at 95% CI and p value <0.05 as statistical significance.

**Results:**

Out of 246 students, 221 fulfilled inclusion criteria. Male to female ratio was 1:3. Mean age was 21 years (S.D: ±0.927). 96.4% were regularly using electroacoustic devices. 47.9% reported their use over five years. Insert type earbuds (73.8%) were the most preferred. Smartphone being the most common source (90%). Upon PTA, one third of medical students demonstrated sensorineural hearing loss at 0.25kHz and 0.5kHz. 9.5% reported associated tinnitus. Daily listening duration exceeded one hour among 78.8% while 26.4% practiced high volume setting. Males’ average listening duration exceeded that of females (p=0.013). However, their mean audiometric thresholds did not vary significantly.

**Conclusions:**

Mild sensorineural hearing loss was detected among one third of participants using personal listening devices. Precautions should be practiced while using these devices.

## INTRODUCTION

Efficient patient-doctor communication is the doorway to trust building, adherence, and good patient outcomes. Majority of the malpractice cases filed against physicians stem from poor communication and understanding.^1^ The need for effective communication has been intensified in this pandemic when wearing masks has been a must. Concealing all the facial expressions and lip movements assisting in understanding language while giving unspoken clues about patient’s concerns and psychological distress.^2^

The paradigm shift from physical learning to online education has further peaked the use of personal listening devices like headphones, ear plugs and Bluetooth. These devices have potential of generating sounds above 125dB.^3^ Daily noise exposure above 85dB (Permissible Exposure Limit) over a period of eight hours can cause noise induced hearing loss (NIHL).^4^

World Health Organization (WHO) has regarded recreational noise exposure as a great threat to the hearing of young people with about 1.1 billion at risk.^5^ Occupational noise hazards have been evidently defined and protective measures are adopted globally.^6^ But no such preventive methods are clearly devised for the protection of dreadful effects of recreational noise neither taught in medical education. Noise induced acoustic trauma has been conventionally described to effect high tone frequencies when assessed by pure tone audiometry (PTA), a clinical diagnostic test to determine the degree and type of hearing loss. Based on the guidelines of Health and Safety Executive it was proposed that the frequency where notch appears in a pure tone audiogram suggests the specific type of noise to which one was exposed. Intense low frequency noise can cause maximal loss at lower frequencies while intense high frequency sound can predominantly affect higher frequencies.^7^

A large population-based study found that hearing loss considerably affect mental health and quality of life.^8^ After aging, noise exposure either occupational or recreational is the leading cause. Noise induced hearing loss (NIHL) once established, is irreversible, only partly manageable though totally preventable.^9^ Limited data is available for medical students assessing hearing thresholds through objective clinical testing. Through this study we aimed to ascertain the current practices prevalent among medical students regarding electroacoustic devices. We also intended to determine the frequency and pattern of hearing loss among medical students using Pure Tone Audiometry.

## METHODS

### Operational Definition

Electroacoustic devices refer to transducers which convert electrical signal into sound signal e.g., handsfree, headphones, Bluetooth etc.

This cross-sectional study was conducted among medical students at Jinnah Sindh Medical University (JSMU), Karachi. Data collection dated from December 2019 till February 2020. The study was approved by Institutional Review Board (IRB) of JSMU (Ref: JSMU/IRB/2019/-215). Students of 3^rd^ year and 4^th^ year MBBS, either male or female and age between 19-24 years were included in the study. Whereas exclusion criteria comprised of students not giving consent, those with type I diabetes mellitus, acute upper respiratory tract infections, acute or chronic ear infections, allergic rhinitis, positive history for ototoxic drugs, past medical history of childhood meningitis, enteric fever in childhood, past surgical history for cleft lip or palate, using hearing aid and family history of hearing loss.

Non-probability convenience sampling technique was employed for data collection. A related study reported prevalence of 84% hearing loss among mobile phone users.^10^ Using this information in Open Epi calculator at 95% confidence interval (CI) and error of ±5%, sample size of 194 was obtained.

Students were invited to the Ear, Nose and Throat (ENT) OPD, Jinnah Postgraduate Medical Centre (JPMC), Karachi for PTA. After informed consent, through a structured proforma sociodemographic information, electroacoustic device usage history and relevant medical and surgical history was obtained from each participant. Sociodemographic data included age, gender, year of study and residence. We also asked about type of electroacoustic device (insert type earphones, supraural headphones or Bluetooth), per day duration, source to which these devices were connected and using since when.

PTA was performed by trained audiologists in a soundproof booth. Air conduction (AC) was tested at octave frequencies i.e., 250 Hz, 500 Hz, 1000 Hz, 2000 Hz, 4000 Hz and 8000 Hz. To differentiate the type of hearing loss from conductive to sensorineural, bone conduction was assessed. Test frequencies for bone conduction were from 500 Hz to 4000 Hz. Air-bone gap was considered significant when it was greater than 15 dB between air and bone conduction thresholds. Findings of audiometry were generated on an audiogram. WHO grading system of hearing impairment was applied to classify hearing loss. Normal was regarded 25 dB or less, mild hearing loss from 26-40 dB, moderate from 41-60 dB, severe from 61-80 dB while profound hearing loss including deafness was 81dB or greater.

Data entry and analyses were conducted using SPSS Software, version 23 (IBM Corp.). Descriptive statistical analyses were run to obtain the frequencies, mean and standard deviation (S.D.). Chi square test and Fischer exact test were utilized to find the association of electroacoustic device use with independent variables like gender and year of study. Independent t test was used to compare the means at octave frequencies among male and female students. P value <0.05 and 95% CI was kept as level of statistical significance.

## RESULTS

A total of 246 students participated in the study. However, only 221 satisfied the inclusion criteria. Mean age of students was 21 years (S.D: ±0.927; Range: 20-24). Out of 221, 74.7% (n=165) were females while 25.3% (n=56) were males. Students from 3^rd^ year and 4^th^ year were almost equal (49.3% vs. 50.7% respectively). Table-I represents sociodemographic characteristics of the study participants.

**Table I:** Sociodemographic characteristics of the Study Participants.

Total Study Population (N)	221

Characteristics	No. (%)
Mean Age (S.D)	21 (±0.927)
***Gender***	
Male	56 (25.3)
Female	165 (74.7)
***Year of study***	
3^rd^ year	109 (49.3)
4^th^ year	112 (50.7)

Among recruited sample, 96.4% (n=213) were regularly using electroacoustic devices. With insert type earphones being the most common (73.8%; n=163). Followed by Bluetooth (14%; n=31) and supra-aural headphones (5%; n=11). Students reported smartphone (90%; n=199) as the most frequently used source for listening to these devices, followed by laptop (32.6%; n=72) and tablet (5.9%; n=13). Listening duration of 78.8% (n=99) medical students exceeded one hour on regular basis. Nevertheless, 19.5% (n=43) exceeded 3 hours per day. In our study, 26.4% (n=58) undergraduates practiced high volume setting for listening. Near half of the users (47.9%; n=106) were using electroacoustic devices beyond 5 years. Wherein 22.6% (n=24) were enjoying their use for 10 or more years. Listening habits of the study participants are summarized in Table-II.

**Table II:** Listening habits of the study participants.

Total Study Population (N)	221

Characteristics	No. (%)
***Electroacoustic device use since***	
Never	8 (3.6)
1-2 years	35 (15.8)
3-4 years	72 (32.6)
5-6 years	52 (23.5)
>7 years	54 (24.4)
***Volume level***	
No use	8 (3.6)
Low	10 (4.4)
Medium	145 (65.6)
High	58 (26.4)
***Listening time per day***	
No use	8 (3.6)
Less than 1 hour	39 (17.6)
1-2 hours	99 (44.8)
2-3 hours	32 (14.5)
>3 hours	43 (19.5)
***Other symptoms (if any)***	
Tinnitus	21 (9.5)
Vertigo	6 (2.7)
Earache	11 (5)
Headache	39 (17.6)

**Fig.1:**
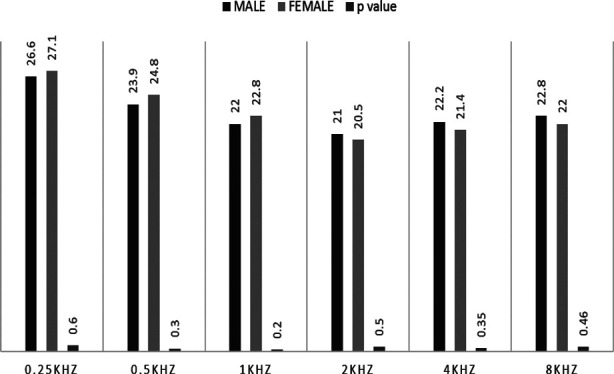
Right Ear Mean Thresholds.

**Fig.2:**
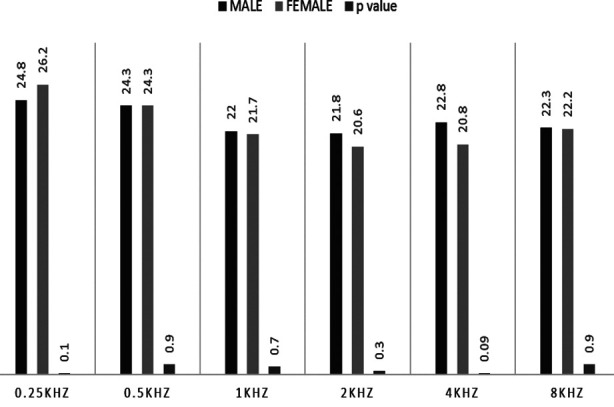
Left Ear Mean Thresholds.

Utilizing independent *t* test carried at 95% CI, statistically significant difference was observed for average listening duration between male (165.77 ± 103.39 minutes) and female students (120.09 ± 121.76 minutes) *t* (219) = 2.516, *p*=0.013. Fig.1 and Fig.2 graphically displays the mean hearing thresholds for right and left ear respectively among both genders.

To detect association between gender and volume setting preference Chi square test was used. However, no significant difference for volume setting (p=0.851) and duration since years (p=0.145) was observed among both genders. Likewise, no statistically significant difference existed between volume setting preference (p=0.977) and duration since years (p=0.820) with year of study.

Audiometric testing demonstrated hearing loss among one third of medical students who were using electroacoustic devices. Sensorineural hearing loss of mild grade (WHO classification) was found at frequencies 250 Hz (31.9%; n=68) and 500 Hz (31.5%; n=67) in right ear. In left ear, 29.1% (n=62) at frequency of 250 Hz while 23% (n=49) at 500 Hz suffered mild sensorineural hearing loss. In this study only 5.6% (n=12) and 6.6% (n=14) students showed audiometric notch at 4kHz and 8kHz in right ear, respectively. Among other symptoms, participants of this study reported tinnitus (9.5%; n=21), vertigo (2.7%; n=6), earache (5%; n=11) and headache (17.6%; n=39). Table-III represents the hearing thresholds of electroacoustic device users at audiometric octave frequencies.

**Table III:** Hearing Thresholds of Pure Tone Audiometry at Octave Frequencies among Electroacoustic Device Users (n=213).

Test Frequency	Normal (<25dB)	Mild HL^1^(26-40dB)	Moderate HL (41-60dB)	Severe HL (61-80dB)
Right ear 250 Hz	139 (65.3)	68 (31.9)	6 (2.8)	-
Right ear 500 Hz	145 (68.1)	67 (31.5)	1 (0.5)	-
Right ear 1000 Hz	198 (93)	15 (7)	-	-
Right ear 2000 Hz	202 (94.8)	10 (4.7)	1 (0.5)	-
Right ear 4000 Hz	200 (93.9)	12 (5.6)	1 (0.5)	-
Right ear 8000 Hz	196 (92)	14 (6.6)	2 (0.9)	1 (0.5)
Left ear 250 Hz	150 (70.4)	62 (29.1)	1 (0.5)	-
Left ear 500 Hz	163 (76.5)	49 (23)	1 (0.5)	-
Left ear 1000 Hz	199 (93.4)	13 (6.1)	1 (0.5)	-
Left ear 2000 Hz	199 (93.4)	12 (5.6)	1 (0.5)	1 (0.5)
Left ear 4000 Hz	198 (93)	13 (6.1)	1 (0.5)	1 (0.5)
Left ear 8000 Hz	194 (91.1)	16 (7.5)	2 (0.9)	1 (0.5)

HL = Hearing Loss.

## DISCUSSION

Our study comprised 221 participants with age group between 20-24 years (M±S.D: 21±0.927). Similar range was mentioned in comparable studies.^11^,^12^ Male to female ratio was 1:3. This represents comparative larger number of female students studying in medical colleges of this region.

A high prevalence (96.4%) of electroacoustic device usage amongst medical students was found. Rekha et al. reported personal listening device (PLD) use by medical students with frequency of 86.1% on daily basis.^13^ A study from Hamadan University of Medical Sciences, Iran stated 91.2% prevalence of PLD use.^14^ A recent study conducted by Basu et al. narrated 5.4% medical students never used an electroacoustic device.^15^ In our study only 3.6% students denied their use of personal listening devices. This high prevalence of electroacoustic device use can be attributed to current educational practices followed by medical students. Such as online lectures and 3D animated content available for vast academic topics.

Participants of our study preferred insert type earphones. The most widely used source was smartphone. Parallel studies observed the similar preferences.^13^,^14^ A study from Jeddah stated that almost all the medical students used a smartphone.^16^ Easy availability of smartphones, comfortable portability along with broad range compatibility for wide variety of earphones are the possible attractions making them the first choice among their users. Near half of our participants were using electroacoustic devices for more than five years. Previous studies reported variable results for association between hearing loss and listening duration.^11^,^12^ Volume preferences did not vary considerably from alike studies.^13^,^14^

Participants of this study demonstrated low frequency of subjective hearing symptoms (tinnitus 9.5%, vertigo 2.7%) in comparison with participants of other studies.^13^,^14^,^17^ Interestingly, we also noted that majority of medical students who displayed hearing loss in PTA were not experiencing tinnitus and even not aware of their declining hearing thresholds. For example, in right ear at 500Hz, 88.4% (n=61) who were having hearing loss did not complain tinnitus.

Upon PTA, around one third of our medical students revealed mild sensorineural hearing loss at lower frequencies (250 Hz and 500 Hz). Similar pattern of low frequency hearing loss was detected in a study conducted among 56 medical students.^18^ A study was performed among 136 employees of a Malaysian telecommunication company. This revealed impaired hearing in 21.2% of the personnel. An equal distribution of hearing loss in low, middle, and high frequencies was noticed.^19^ The possible explanation to this distinctive pattern of low frequency loss might be due to the intensity of noise to which they were exposed as indicated by McBride et al.^7^ The participants of our study were medical students who might be using electroacoustic devices for educational purposes mostly. The staff of telecommunication company used headphones for receiving phone calls which involve conversational frequencies. The intensity, pitch and bandwidth of sound generated in such content differ considerably from that produced in music and occupational noise. In addition, a prolonged exposure up to eight hours per day over 85dB is required to produce this characteristic pattern.^4^ None of our study participants reached this limit hence traditional notch at frequency of 4kHz was not found among most of the users.

In our study we found mild sensorineural hearing loss (26-40dB) in majority of cases. This ‘mild’ degree of hearing loss, however, does not accurately implies the functional limitation. PTA does not measure supra-thresholds deficits including frequency selectivity, temporal resolution, and pitch perception, etc. All are which functional components and necessary for speech understanding. Loss of frequency selectivity poses difficulties in understanding speech in a background noise. Temporal resolution deficits make hearing of consonants and vowels difficult. While deficient pitch perception creates problem in identifying prosodic aspects of speech (differentiating a statement from a question), recognition of speaker and difference of speech sounds.

Effect of ‘mild’ hearing loss is significantly larger on communication. About half of the audible information at conversation-level speech will be missed by a person having ‘mild’ hearing loss. This amount is increased for quite speech or a distant level speech. A ‘moderate’ degree hearing loss distorts the conversational-level speech even at close range. While ‘severe’ hearing loss will render a person inaudible of close speech.^20^ Thus, degree of hearing loss is not indicative of perception deficits, communication defects and quality of life.

### Limitations

Present study has certain unavoidable limitations owing to its study design. Causal relationship cannot be confirmed based upon findings of this study for which experimental studies are required. This was a single centre study conducted through convenience sampling hence findings cannot be generalized.

## CONCLUSION

This study revealed mild sensorineural hearing loss among one third of participants. This is worrisome and necessitates inclusion of targeted preventive medicine modules in medical curricula regarding modern technology and their health effects. As this type of hearing impairment once established, is irreversible but completely preventable.

### Authors’ Contributions:

**SA:** Conception of study topic, formulation of questionnaire, data collection, data entry and analysis, drafting of manuscript

**HK:** Formulation of study design, data collection, clinical relevance, interpretation of results

**SP:** Data collection, data entry and drafting of manuscript

**SMTR:** Provision of study subjects, interpretation of results, final review of draft for important intellectual content.
